# Closing gender gaps through gender-responsive, demand-led breeding in Burundi

**DOI:** 10.3389/fsoc.2023.1264816

**Published:** 2024-01-05

**Authors:** Blaise Ndabashinze, Eileen Bogweh Nchanji, Cosmas Kweyu Lutomia, Eric Nduwarugira, Marie Bernadette Hakizimana, Immaculée Mayugi

**Affiliations:** ^1^Institut des Sciences Agronomiques du, Bujumbura, Burundi; ^2^International Center for Tropical Agriculture, Nairobi, Kenya

**Keywords:** bean breeding, gender equity, gender-responsive breeding, Burundi, traders, value chain, demand-led breeding

## Abstract

Gender inequality persists in Burundi’s agricultural sector, especially in the bean value chain dominated by women. Women often have less access to improved seeds and to productive technologies. Interventions dubbed “gender-responsive plant breeding” have been launched to develop new varieties to address the gender gaps in variety adoption. Gender responsive planting breeding in Burundi targets to develop bean varieties that respond better to gendered varietal and trait preferences. This paper provides a background of gender-responsive bean breeding in Burundi, documenting the methodologies that were used to integrate gender issues in bean breeding and socio-economic research. It also covers successes of gender-responsive breeding to date, primarily focusing the interdisciplinary teams that drove the process, development and release of varieties that incorporated traits favored by women and men actors. Evidence from surveys and value chain analysis reveal that gender-responsive breeding program increased the adoption of improved varieties by women and improved yields and productivity. The paper reveals that gender-responsive and demand-led bean breeding programs require stakeholders engagements to develop products that align with preferences of diverse actors at different nodes of the bean value chain.

## 1 Introduction

### 1.1 Why did the breeding initiative pay attention to gender?

Common bean is one of the most widely produced, traded, and consumed leguminous crops in sub-Saharan Africa. Important dynamic in common bean production, trade, and consumption is the dominant role of women. Therefore, bean breeding programs have for the past decade paid attention to gender issues in the bean value chain. Even though breeding programs are important tool for improving food security and nutrition in sub-Saharan Africa (Kondwakwenda et al., 2022), they need to take gender issues into account to address social issues – gendered needs and preferences of end users and gender-differentiated adoption and outcomes – associated with technology adoption (Tufan et al., 2018). Gender considerations in plant breeding ensures that breeding products responds to diverse needs and priorities of men and women farmers, traders, processors, and consumers. Traditional gender roles and relations in Burundi favour men’s effective participation in agriculture (Rames et al., 2017). Gender roles in Burundi’s bean value chain are clearly defined. Men are primarily responsible for managing land and agricultural inputs. In contrast, women typically provide farm and post-harvest labour in bean production. Women are heavily involved in labour-intensive activities, such as land preparation and weeding and harvesting and post-harvest activities. Despite their contribution to bean production. Household power dynamics in Burundi’s farming households often excluded women from decision-making in agriculture, limiting their access to resources and technologies (e.g., land, seed, and other farm inputs) as men, and prevents them from benefiting from their participation in bean production (Nchanji, 2020).

The significant participation and contribution of women in bean value chain in Burundi informed breeding initiatives’ attention to gender. While there is substantial women’s presence in Burundi’s bean sub-sector, there are limited comparative studies that capture gender dynamics and existing gender gaps in use of bean technologies (Nchanji et al., 2023a). Biofortified bean breeding initiatives also paid attention to gender to address malnutrition and food security issues among rural households (Nchanji et al., 2023b). Women in Burundi play a crucial role in household nutrition and food security and therefore their involvement and consideration in bean breeding programs was vital for effective tackling of malnutrition. As noted by Nchanji (2020), women benefit less from participation in the bean value chain compared to men. Thus, breeding initiatives in Burundi paid attention to how gender integration can contribute to women’s empowerment, increased access to resources, and economic benefits. Finally, breeding initiatives attention to gender was also a culmination of the recognition of the need to make breeding programs inclusive and effective by considering diverse needs and preferences of all stakeholders as emphasized by breeding programs elsewhere in Eastern and Southern Africa (Nchanji et al., 2022).

## 2 Context

The team that worked on gender responsive breeding was drawn from the Institute of Agricultural Science of Burundi (ISABU) and the Alliance of Bioversity International and CIAT (ABC). The ISABU team was composed of two bean breeders, a gender expert, two agronomists, and one socio-economist. The ISABU team was supported by experts from ABC through the Pan-Africa Bean Research Alliance (PABRA) programs in Burundi. The ABC team consisted of a gender and social Inclusion expert, an integrated crop management (ICM) expert, a regional bean breeder, seed systems and impact assessment experts, a nutritionist, and a socio-economist. The breeders owned the breeding process and made decisions about bean traits, guided by information from team experts.

The role of common bean in addressing food and nutrition problems and empowering women in Burundi was recognized by the Burundian government four decades ago. As the government, through ISABU, initiated several bean breeding programs in response to government commitment food security and nutrition and women empowerment commitment (PABRA, 2021). The breeding programs were further strengthened after the establishment and commencement of PABRA activities in Burundi in 1998. The International Center for Tropical Agriculture (CIAT)- supported PABRA in early 2000 to work with ISUBU Burundi to rebuild it bean research programs following years of civil war which culminated in launch of a flagship project - Improving food security, nutrition, incomes, natural resource base and gender equity for better livelihoods of smallholder households in Sub-Saharan Africa – in 2015 the project focused on improving food security, nutrition, incomes, natural resource base, and gender equity. This project aimed to strengthen seed production and increasing women’s access to production resources.

The journey towards gender-responsive bean breeding in Burundi’s bean breeding programs can be grouped into three periods: before 2015, 2015 to 2018, and after 2018. Before 2015, breeding programs in Burundi were driven by breeders, focusing on farmers’ varietal and trait preferences, with no consideration of gender. Additionally, breeders paid little attention to the interests of traders, processors, and consumers, who were not invited to the PVS studies. Data was collected solely by breeders and agronomists, and traits were ranked to reflect farmer preferences.

A series of awareness raising events on gender in breeding took centre stage in the 2015–2018 period, which was marked by increased attention to gender. The gender expert from ABC made three presentations on the importance of gender integration in participatory varietal selection (PVS) during different meetings in Burundi. The ISABU team received training in integrating gender in PVS and other breeding activities. Breeders and agronomists attended four regional breeding meetings in Tanzania, Kenya, Ethiopia, and Uganda where gender integration was intentional. The most recent training happened was May 2023 in Tanzania. Furthermore, PABRA sponsored ISABU agronomists and breeders to attend a training in Nairobi, where other value chain experts were invited. The meeting oriented the ISABU team to widen its interest in the bean value chain.

PABRA also sponsored ISABU breeders and agronomists to attend the Gender-Responsive Equipped Agricultural Transformation (GREAT) training in Uganda, where breeders from several African countries shared experiences about integrating gender in breeding and seed systems, including challenges, solutions, and opportunities. The GREAT training was an eye opener for the Burundi breeders about entry points for gender-responsive bean breeding. For instance, the GREAT training enabled ISABU breeders and agronomists to recognize opportunities for gender-responsive breeding, including benefits for targeting smallholder women farmers, entrepreneurs, and farmer organizations, in increasing use of plant breeding products. The training also enhanced the capacity of ISABU breeders and agronomists in designing research projects that maximize delivery of equitable outcomes for all genders.

During the 2015–2018 period, PABRA gave financial support to the ISABU team to collect data with a gender lens. This support led to two publication of research papers on gender dynamics and social norms in bean production among smallholders in Burundi (Nchanji et al., 2023a,b). A stakeholders’ meeting was also organized in Burundi, attended by bean traders and processors, to enable the ISABU team to understand that crop variety and traits preferences are not uniform, but vary across gender, sociocultural contexts, agro-ecological factors, and type of stakeholder (Nchanji et al., 2023b). During the workshop, the ISABU team created a common understanding of demand-led breeding, which not only concerned gender equity, but also inclusivity. The ISABU gender expert became part of the data collection team to ensure that PVS was not farmer-centric, but included data collected from men and women farmers, traders, processors, and consumers.

After 2018, all team members assumed clearly defined roles and responsibilities. Breeders were still dominant, but other team members now helped to guide breeding objectives, and to give greater attention to demand-led breeding. The ABC gender expert trained the ISABU team on how to integrate the gender product profile tool (G+ tool) with existing data collection tools, such as PVS (Nchanji et al., 2022). The G+ tools, initially adapted for gender-responsive PVS in Kenya (Nchanji et al., 2021), were further customized and applied to similar breeding programs in Burundi. Communication between gender specialists, the socio-economist and breeders became frequent. The ISABU breeders currently use information from gender analysis and market studies in product advancement decisions. Bean breeding in Burundi are now focuses on multiple themes, including end-user traits, yield potential, and tolerance to biotic and abiotic stresses (Mukankusi et al., 2019).

The breeders’ takeaway messages from the training were that breeding objectives should focus on the needs of traders, processors, and consumers, and not just on men and women farmers. Breeders also learned to align breeding objectives with the national development goals, including nutrition and health, and equality. The breeders and agronomists appreciated and understood different roles, responsibilities, and needs of women and men in the bean value chain, the importance of inclusive design processes of breeding programs, development of gender-sensitive research methodologies, and importance of building networks and collaborations with other researchers and institutions focused on gender-responsive agricultural research.

## 3 Analysis

### 3.1 What research or other sources of information on gender were generated?

After the GREAT training, ISABU conducted gender study to understand gender differences among different actors and to provide evidence for gender-responsive breeding. The data were collected in 2019 using several methods. The gender-responsive PVS tool collected socioeconomic and varietal and trait preferences of men and women farmers. Gender gap questions from the G+ Product Profile tool was adapted and incorporated into the PVS to capture the social implication of farmers’ preferred varieties and traits. The incorporation of the gender gap questions was based on experiences in gender-responsive breeding by PABRA in biofortified bean varieties in Zimbabwe (Nchanji et al., 2021). For instance, they asked if the preferred traits increased or reduced drudgery for women, and if the best traits changed women’s access to inputs or influenced women’s control of benefits and income.

Sex-disaggregated PVS data that also collected information on men and women varietal and trait preferences were used to inform breeding decisions, especially which varieties and traits to prioritize for different actors and bean regions. PVS results indicated that it was nice to have additional traits beyond the biotic and abiotic stress traits. For instance, contrary to the expectation that women would rank cooking time among the”first best“traits, they ranked it fourth. While men ranked price and marketability of the beans as the reason for choosing the top-ranked or best variety, women emphatically preferred high yield. The PVS data also shed light on the preferences of different stakeholders, such as consumers. Consequently, breeders deliberately used the PVS varietal and trait preferences data to define breeding objectives and strategies, and to develop new varieties that met the needs of the local men and women farmers.

ISABU and CIAT also conducted social surveys to understand bean production in local contexts. In the first study, Nchanji et al. (2023b) collected and analyzed data on gender dynamics in Burundi’s biofortified bean value chain to illuminate the roles of men and women in the bean value chain. The data also highlighted how marital status, education, source of seed, land area cultivated, pesticide use, household income, hired labour, and gender of the household head influenced farmers’ demand for extension, a critical driver of adoption of bean varieties. The second social survey study focused on the implications of social norms on gendered differences and access to climate-smart agricultural technologies and the gains in closing the gender gaps through gender-responsive plant breeding (Nchanji et al., 2023c). The study highlights that gender-responsive plant breeding has resulted in gender-responsive systems that now recognize challenges, interests, and preference of women. Nchanji et al. (2023c) acknowledges that gender-responsive breeding in Burundi has positively influenced women farmers use of certified seeds and allocation of land under bean production for women.

The interdisciplinary team from ABC and ISABU also conducted value chain analysis with traders, processors, and consumers to inform bean breeding (PABRA, 2021, 2022). Meeting with traders and processors collected information on the importance they place on different traits and the criteria they use to evaluate bean varieties. A consumer study in 2020 assessed the uptake of bean products (flours) produced by processors supported that were supported by PABRA program that aimed scale up production of composite bean flour for weaning infants and lactating mothers using biofortified beans (PABRA, 2021). The data helped to explain the uptake of nutrient-dense food products promoted by PABRA. The consumer data also provided insights on the potential demand for processed bean-based foods and drivers of demand for these products, made from bean varieties developed by the gender-responsive breeding program.

### 3.2 How attention to gender influenced the breeding initiative

Attention to gender has influenced the gender breeding pipeline in Burundi in several ways. First, gender-specific approaches ensured that the breeding initiative was not only in line with ISABU’s objectives, but also those of actors in bean value chain. The breeding team’s attention to gender ensured that future bean varieties would consider farmers’ gendered traits. The breeders’ attention to gender also helped to prevent any issues that could arise from a poor understanding of social issues. These influences can best the illustrated through a narrative of what occurred before and after the GREAT training in 2019.

The ISABU team was encouraged by the gender expert at the Alliance of Bioversity and CIAT to attend the GREAT course on gender-responsive breeding. The training acknowledged that despite the contribution of breeding in last five decades, most efforts targeted higher yield and resistance to climate change, pests, and diseases. The training highlighted how gender-responsive breeding is increasingly becoming popular and its implications on the genetic diversity of crops and how they address social issues (Polar et al., 2022). They described gender-responsive breeding as research that helps solve social problems such as food insecurity, poverty, and gender inequality by considering the social contexts where farming takes places and developing new bean varieties that are better suited to the needs of men and women.

The ISABU breeders and the social scientists learned that men and woman had different roles that affected their choice of varieties and traits. Additionally, the gender roles had to be continuously considered if the breeding products were to be inclusive. These realizations precipitated the inclusion of women farmers, traders, and consumers in the design stage of bean breeding. The Burundi bean research team realized that this was essential for the success of the plant breeding because of the crucial role played by women. They realized that women have unique perspectives of production and entrepreneurship problems and solutions that are often overlooked by conventional breeding. The ISABU breeders and economists understood that women are often more attuned to the needs of local communities, which can help breeding programs successfully address social issues in adoption and use of breeding products. Including women in plant breeding created a more diverse environment for better outcomes for everyone.

After the GREAT training, the ISABU team gave greater attention to gender, refining the PVS tools that breeders had previously used. Rather than relying on PVS to identify potential varieties for release, breeders at ISABU, with valuable contribution from gender expert who was previous not involved in bean breeding programs, incorporated the “gender-responsive PVS” tool into plant breeding programs, to identify traits that meet the needs of men and women farmers and traders. In addition to sociodemographic questions, the PVS tool asks the gendered implications of “best traits” on drudgery, demand for inputs, and women control over benefits and/or income from bean production (Ashby and Polar, 2021) Learnings about gendered preferences were incorporated in biofortified bean breeding programs in the country.

The ISABU breeding team’s attention to gender also enabled them design new bean varieties that captured cooking time, nutrition, ease of threshing, and organoleptic traits in addition to yield, drought tolerance and pest resistance traits that were targeted by breeders before 2015 (Table 1). The gender-responsive breeding team used organoleptic tests to understand consumers’ preferences, and helped breeders evaluate the quality of the varieties they in the breeding pipeline. This organoleptic evaluation assessed taste, smell, texture, and the appearance of processed products, such as boiled beans, flour, cakes, porridge, and snacks. ISABU breeders identified desirable traits and used them to create new varieties that appealed to consumers, especially women who are responsible food preparation and household diets. Organoleptic testing also helped breeders identify any potential problems with new varieties, such as off-flavours or discoloration, which were to be addressed before variety release. Certain traits like short cooking time and biofortification with zinc and iron gained traction.

**Table 1:** Traits considered in bean breeding in Burundi before and after gender-responsive breeding.

Traits considered before 2015	Traits considered between 2015 and 2015	On-going
Drought tolerance	Drought tolerance	Drudgery
Resistance to pests and diseases	Resistance to pests and diseases	Pod shattering
High yielding	High yielding	
	Nutrition	
	Ease of threshing	
	Input requirements	
	Taste	

## 4 Methods and approaches: advantages and shortcomings

The bean breeding team in Burundi learned that men and women have different preferences for bean varieties, which reflect gender roles and inequalities. Bean breeding programs before 2015 were not gender-intentional when analyzing farmers’ trait preferences. Breeders were farmer-centric, designing products that they thought would be suitable for farmers in general. Participatory variety selection (PVS) were typically done through general conversations with a few farmers. After 2015, several methods of data collection started to be used to collect social, economic, and breeding data (Table 2). Breeding initiative paid attention to gender after several PVS exercises (with about 50 to 60% female participation) revealed that men and women had different trait preferences. Women’s increased participation in PVS was a recognition of their role in bean production and household food security. Women were recognized as vital for agricultural development, and their access to preferred, locally-adapted varieties was important in increasing yields, improving household nutrition, and promoting the equitable distribution of benefits.

**Table 2:** Methods used to generate more evidence on gender gaps and increase women’s participation in bean breeding in Burundi.

Activity	Attention to gender	Method
Participatory Varietal Selection (PVS)	Yes	Intentional/targeted involvement of more women
Social survey research – gender analysis	Yes	Random and purposive selection of farmers using an intersectional perspective from multiple farmer groups
Consumer and trader studies	Yes	Direct interviews with key diverse respondents and focus group discussions during organoleptic tests. Key informant interviews and informal discussions in stakeholder meetings Gendered quantitative surveys
Value Chain Analysis	Yes	Qualitative value chain mapping through diverse and mixed focus group discussions and quantitative data collection farmers from farmers, traders and processors.

The GREAT training, adoption of value chain analysis as a driver of breeding, and social surveys that documented gender gaps with implications for variety design also shaped attention to gender-responsive anddemand-led breeding in Burundi. These experiences not only exposed breeders to the social and market dimensions of breeding for gender equality but also emphasized that an effective, gender-sensitive breeding program must cater to a spectrum of stakeholders such as traders, processors, consumers and other actors, not just farmers. For instance, the gender expert at ISABU was never involved in the bean breeding program, but following the GREAT training, she took participated in designing the PVS tools (Table 3) and, she became part of the analysis team. Adopting the demand-led, client-oriented breeding approach promoted by PABRA created a fertile ground for the social survey research that strengthened the case for gender-responsive breeding. National food and nutrition security and gender policies implemented by the Ministry of Agriculture were also critical drivers of gender-intentional breeding.

**Table 3:** Preliminary results of PVS data.

Top reason reason for selecting a trait? (%)	Female (*n* = 45)	Male (*n* = 35)
Price	33	38
Yields	36	29
Biofortified – this means “nutritional value” to the farmers?	22	13
Cooking time	4	11
Grain color	4	5
Seed quality	4	2

### 4.1 What in the breeding process and practice has changed because of learning about gender?

The plant breeding process and practice has changed significantly due to increased awareness of gender and social uses. Since 2019, the ISABU team has a gender analyst who is included in team meetings and contributes to decisions about which traits are important and which varieties to release. Bean breeders are now more attentive to the needs of different genders, ethnicities, and communities when they are developing new varieties. This has led to more attention being paid to the nutritional and health benefits of new varieties, as well as the potential for varieties to be used in different cultural contexts. Plant breeders are now more aware of the potential implications of their work on social issues, and they are working to develop varieties that result in positive outcomes for all stakeholders.

### 4.2 Breeding outcomes and impacts, especially those related to the impact on gender equity

The gender-responsive breeding initiative released three bean varieties to meet the needs of smallholder farmers between 2019 and 2021. The development of the varieties MAC52, MAC54, and BFS18 incorporated insights on gender differences in trait preferences. The varieties are fast-cooking and rich in iron and zinc, and they taste better. They are adapted to local diverse agro-ecologies, making them well-suited for smallholder farmers. The release of these varieties enabled men and women smallholders to access superior bean varieties with the potential of increasing yields and incomes, while eliminating gender inequalities along the bean value chain. With access to varieties that are better adapted to their local conditions, women farmers can produce more with less effort and resources. The improved nutritional properties of the varieties may improve the diets of rural communities, while helping to reduce food insecurity and social inequalities.

The gender dynamics study in 2021 indicated that 60% of farmers (62% women and 57% men) had adopted the MAC44 bean variety in Muyinga and Gasorwe communes of Muyinga province (Nchanji et al., 2023c). The second study showed that gender-responsive activities in Burundi closed gender gaps in the adoption of improved bean varieties, with more women adopting improved seed than men. 75% of men and young farmers used improved varieties compared to 85% of women farmers in Kirundo and Bwambarangwe communes in Kirundo province and Muyinga commune in Muyinga province (Nchanji et al., 2023c). The adoption rates are high because the communes were ISABU and PABRA program sites. The adoption has improved productivity resulting in increased income, food security, nutrition, and health. These positive impacts have been greater for women than for men. From the impact study in 2020, men and women who planted improved bean varieties recorded a 40% increase in bean yields. Partial (planted both improved and local varieties) adopters improved their bean yields by 27%. The greater bean yields resulted in 61% higher incomes for men and women, and a 15.8% increase in bean profitability (Katungi et al., 2020).

The importance of gender-responsive breeding is now increasingly being recognized in Burundi. There is increasing understanding by breeding teams, agronomists, socio-economists, and gender experts that breeding should not only recognize the differentiated roles and needs of women and men in agriculture but also equitably benefit all genders and actors in the bean value chain. For instance, plant breeders are now paying more attention to the potential of new varieties to provide multiple benefits to different social groups, such as providing food security and economic opportunities to women. The breeding initiatives are also now targeting to contribute to women empowerment and by not only developing products that captured men and women trait preferences but also contributing to addressing inequalities by generating products that improve agricultural productivity and economic benefits at different nodes of the bean value chain. Such gender-centred initiatives promises to break cultural, social, and economic barriers that constrain women participation in bean value chain beyond production and informal trade.

## 5 Good practices and lessons

### 5.1 Good practices

The gender-responsive bean breeding initiative in Burundi generated multiple good practices that are critical for similar future initiatives. Key among these is the all-inclusive engagement of stakeholders, including women farmers, traders and consumers, from design to implementation which ensures of breeding programs, to ensure that breeding objectives are aligned with needs and priorities diverse groups. Another best practice is that interdisciplinary approaches to breeding are required to enable mainstream breeding teams and social scientist to contribute their knowledge to enrich the breeding process. Additionally, trainings such as GREAT course are instrumental in enhancing breeding team’s understanding and implementation of gender-sensitive approaches in breeding programs. The development and adaptation of data collection tools to local contexts, including the gender responsive PVS, helps in the identification of the traits, which were important not only for men but also for women. Commitment to data-driven decision-making also helped to effectively address gender gaps.

Another important lesson drawn from gender-responsive plant breeding in Burundi is that balancing abiotic and biotic traits with traits like cooking time and nutritional value that is particularly very important to women is critical. Furthermore, continuous monitoring is needed to assess the gender impact of gender-responsive breeding to ensure initiative’s alignment with broader national policies on food security, nutrition, and gender equity. Finally, gender-responsive breeding initiatives in Burundi demonstrated the need for scalability and sustainability in similar projects.

### 5.2 Lessons

The gender-responsive initiatives in Burundi in the last decade offer important lessons for agricultural innovations. First, there should a strong emphasis on integrating gender considerations in agricultural research and development to cater for diverse needs and priorities for effective and inclusive innovations and outcomes. Second, interdisciplinary approach, collaboration of expertise from agronomists, gender experts, socio-economists, and breeders, is pivotal for the success of gender-responsive breeding. Collaboration during breeding is crucial in integrating skills and nuanced perspectives to the intersection of agriculture and gender. Key to the effectiveness of the gender-sensitive breeding was training and capacity building, as demonstrated by the GREAT program. Specifically, training was essential for creating gender-responsive awareness among breeders and agronomists. Another important lesson is that alignment breeding program initiative with national and regional policies on food security, nutrition, and gender equity is paramount for sustainable impact. Finally, assessment of gender impact of breeding programs and addressing emerging challenges that may prevent inclusive adoption are essential for the success of gender-responsive agricultural initiatives.

## Data availability statement

The raw data supporting the conclusions of this article will be made available by the authors, without undue reservation.

## Ethics statement

The studies involving human participants were carried out by ISABU - a government entity - in accordance with the local legislation and institutional requirements. The participants provided their verbal and written informed consent to participate in this study.

## Author contributions

BN: Conceptualization, Formal analysis, Funding acquisition, Methodology, Project administration, Visualization, Writing – original draft, Writing – review & editing. EBN: Conceptualization, Formal Analysis, Funding acquisition, Methodology, Visualization, Writing – original draft, Writing – review & editing. CL: Formal Analysis, Methodology, Writing – review & editing. EN: Data curation, Supervision, Writing – review & editing. MH: Data curation, Supervision, Writing – review & editing. IM: Data curation, Supervision, Writing – review & editing.
